# The Potential Relationship Between Low Back Pain and Depression: A Comprehensive Review

**DOI:** 10.1002/brb3.71026

**Published:** 2025-11-05

**Authors:** Li Cao, Biao Deng, Xiaole Wang, Ting Fang, KuyikAbasi Nsima Umoh, Lang Liu, Jiaming Qian, Fushui Liu

**Affiliations:** ^1^ Jiangxi University of Chinese Medicine Nanchang Jiangxi Province China; ^2^ The Affiliated Hospital of Jiangxi University of Chinese Medicine Nanchang Jiangxi Province China

**Keywords:** AI, brain, depression, hypothalamic‐pituitary‐adrenal (HPA), low back pain

## Abstract

**Purpose of Review:**

The primary objective of this review is to explore and elucidate the potential relationship between low back pain (LBP) and depression. As both conditions are prevalent health issues affecting a significant portion of the population, understanding their interplay is crucial for developing effective treatment strategies. By examining the existing research, this review aims to provide insights into the biological, psychological, and social factors that contribute to this intricate relationship.

**Recent Findings:**

Recent research has shown an interdependent relationship between LBP and depression. Chronic pain can lead to depression by affecting daily life and work, whereas depression can exacerbate pain perception due to alterations in brain processing. Studies have shown that pain and emotional regulation share the same brain regions, providing a neurobiological basis for their coexistence. Furthermore, psychological factors such as anxiety, stress, and poor coping mechanisms have been shown to have associations with both conditions.

**Summary:**

The relationship between LBP and depression is complex and multifaceted. Addressing both the physical and psychological aspects of these conditions is essential for effective management. Interdisciplinary approaches, including pharmacological treatment, psychological therapy, physical therapy, and lifestyle modifications, should be considered to improve outcomes for patients suffering from both LBP and depression.

## Graphical abstract

This work reveals bidirectional neurobiological links between low back pain (LBP) and depression through shared stress pathways. Chronic LBP triggers depression via HPA axis dysregulation and neuroinflammation, whereas depressive states amplify pain sensitivity through neural plasticity changes, establishing a self‐perpetuating comorbidity cycle requiring integrated treatment.

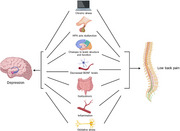

## 1 Introduction

This article was written using the Ernie Bot Large Model 3.5 version. The research, writing, and final editing of the article were conducted by Cao Li and Deng Biao, whereas Liu Fushui conducted an overall review and correction of the article. In the process of utilizing AI writing, this article referred to the BRTR principles mentioned in Margetts, Tyler J.’s article (Margetts et al. 2024), namely, B (Background), R (Role), T (Task), and R (Requirement). The purpose of this article is as follows: first, to summarize recent research on the comorbidity of low back pain (LBP) and depression, as well as broaching potential comorbidities between LBP and depression, hence providing ideas for future research; second, to explore the possibilities of AI writing through the application of AI writing methods.

## 2 Background on LBP

In the field of modern medicine, LBP has emerged as a significant health concern, containing a complex web of biological, psychological, and social factors (Knezevic et al. 2021). It is described as pain or discomfort localized below the rib margin and above the gluteal fold, which may or may not radiate to the legs. This pain can either be axial lumbosacral, radicular, or referred in origin (Urits et al. 2019; Andersson 1999). The epidemiological profile of LBP is alarming; it is a prevalent condition, affecting up to 80% of individuals at some point in their lives, with approximately 18% of the population experiencing it at any given time. In the United States of America (USA), the lifetime prevalence among adults is as high as 65%–80% (Urits et al. 2019), with studies indicating a worldwide estimate of about 619 million people having suffered from LBP in 2020, equivalent to nearly 10% of the global population. This figure is predicted to climb up to 843 million by 2050 (Koppenaal et al. 2023). On the basis of statistical data, the societal and economic burden of LBP is substantial (Carregaro et al. 2020; Shiri et al. 2019). In the United Kingdom (UK), LBP costs the National Health Service (NHS) nearly £5 billion annually from general practitioner appointments alone, and in the US, the expenditure on low back and neck pain was $134 billion in 2016 (Koppenaal et al. 2023). The prevalence of LBP is highest among working‐age individuals, resulting in increased absenteeism, decreased productivity, and early retirement. In Brazil, between 2012 and 2016, LBP accounted for 100 days absent from work per person per year, with productivity losses constituting nearly 80% of the country's annual cost of LBP (Koppenaal et al. 2023).

The specific detriments of LBP are multifarious. Chronic LBP often triggers emotional disturbances and cognitive impairments, significantly affecting the quality of life (Felício et al. 2022). Lifestyle and physical workloads are known risk factors for LBP and lumbar radicular pain, whereas walking and cycling may offer preventive outcomes (Pepin et al. 2024). Notably, depressive symptoms have been identified as a risk factor for back pain, highlighting the intricate synergy between physical and mental health. Negative expectations have been shown to predict poor pain outcomes, suggesting that pain perception is influenced by emotional, cognitive, and behavioral variables (Kandola et al. 2019). A study of adults in Afyon, Turkey demonstrated that elevated BMI (*p* < 0.001) and depression (*p* = 0.016) are significant risk factors for LBP (Altinel et al. 2008). A cross‐sectional study in Punjab, Haryana, and Chandigarh, India, revealed that LBP patients exhibited a mean DASS‐21 depression score of 11.87 (SD = 4.05), with significantly higher scores in females (*p* < 0.05). Among 698 LBP patients, 515 (73.8%) presented mild or greater depressive symptoms (Asrar et al. 2021).

Furthermore, chronic LBP has been associated with structural changes in the brain. Studies have revealed alterations in white and grey matter regions, indicating that chronic pain is accompanied by structural reorganization in the brain (Knezevic et al. 2021). These brain changes are markedly linked to the onset of depressive disorders, providing a biological ground for the potential relationship between LBP and depression.

In conclusion, the complex relation between LBP and depression remains an under‐researched area. Given the significant impact of both conditions on individual well‐being and societal costs, there is a pressing need for more in‐depth research and understanding of this association.

## 3 Background on Depression

Depression, a prevalent mental health issue, is characterized by persistent low mood, dysphoria, impaired motivation, and several other symptoms, ranging from psychomotor to cognitive impairments (Beurel et al. 2020). According to the World Health Organization (WHO), depression affects around 300 million people globally and is now the leading cause of disability worldwide (Beurel et al. 2020). The lifetime prevalence of major depression in the US is estimated to be 21% for women and 11%–13% for men, indicating a clear gender disparity (Shorey et al. 2022). Among adolescents, the point prevalence of elevated depressive symptoms increased from 24% between 2001 and 2010 to 37% between 2011 and 2020. Regionally, the Middle East, Africa, and Asia have the highest prevalence of elevated depressive symptoms, with female adolescents reporting a higher incidence than males (Maier et al. 2021). Additionally, depression is also common in the elderly population (GBD 2016), with meta‐analyses finding prevalence rates of depressive manifestations to be 17.1% in individuals ≥75 years old and 19.5% in those 50 years and above (Diniz 2018). These statistics emphasize a varying incidence of depression not only across age but also across gender.

Depression not only increases susceptibility to other disorders but also hinders treatment outcomes for medical conditions. Depressed patients often experience poorer health outcomes, contributing to a higher disability rate (Malhi and Mann 2018). This condition incurs significant economic burdens due to lost productivity, medical expenses, and societal repercussions associated with decreased functioning (Pinheiro et al. 2016). The risk factors for depression are diverse and include a family history of depression (approximately 35% of the risk is hereditary), early life abuse and neglect, female gender, and recent life stressors. Medical illnesses, particularly metabolic (e.g., cardiovascular disease) and autoimmune disorders, also elevate the risk of depression (Shorey et al. 2022; Pinheiro et al. 2016). It is essential to recognize these risk factors in order to implement preventive measures and early interventions.

Importantly, research has confirmed that depression can negatively impact the outcomes of LBP (Ostir et al. 2002). Studies show that depressive symptoms intensify the perception and sensation of pain, leading to poorer recovery and increased ailment (Tsuji et al. 2016). Recent data from animal models provide neurological evidence that suggests that the regulation of dopamine activity in the ventral tegmental area (VTA) mediates depressive and anxiogenic responses, thereby linking depression and chronic pain (Fu et al. 2016).

Given this intricate relationship, it is necessary to explore the potential pathways between depression and LBP. Below, we will combine the existing research in order to clarify these connections, highlighting the complex interaction between psychological and physical health.

## 4 External Stress and Emotional Imbalance

### 4.1 Chronic Stress Triggers

Chronic stress refers to the persistent activation of the stress response system in the body due to prolonged exposure to psychological or environmental pressures, without adequate rest and recovery (He et al. 2024). This sustained state can lead to physiological and psychological health issues, including neuroendocrine disruptions and decreased cognitive function (Marin et al. 2011). Various stressors, particularly those experienced during childhood, have been shown to increase the risk of developing mental illnesses, such as affective and anxiety disorders. From an epidemiological perspective, chronic pain, especially LBP, is a significant source of chronic stress. The widespread prevalence contributes to a substantial burden on both individuals and society, often leading to reduced quality of life, work incapacitation, and significant healthcare costs. The mechanism through which LBP generates chronic stress is varied. Pain and discomfort can trigger psychological responses, such as anxiety, fear of movement, and concerns about the future, all of which contribute to sustained stress levels. Additionally, the impact of LBP on daily activities and social interactions can increase stress, creating a vicious cycle of pain and stress. Moreover, chronic stress has been associated with the development and progression of depression. Studies have shown that chronic stress can promote the pro‐inflammatory action of glucocorticoids and disrupt the balance of gut microbiota, such as reducing the number of *Lactobacillus* and *Bifidobacterium* species (Góralczyk‐Bińkowska et al. 2022).

Animal models of chronic stress–induced depression have been instrumental in understanding this relationship. To demonstrate, the chronic mild stress (CMS) model exposes animals to a series of unpredictable stressors, leading to behavioral changes akin to those observed in human depression, including anhedonia—a lack of interest or pleasure in activities (Willner 2016). The mechanism by which chronic stress leads to depression involves complex interactions between the hypothalamic–pituitary–adrenal (HPA) axis, neurotransmitters, and inflammation. Under stress, the HPA axis is activated, resulting in increased release of corticotropin‐releasing hormone (CRH) and arginine vasopressin (AVP) (Mitrea et al. 2022). These hormones stimulate the secretion of adrenocorticotropic hormone (ACTH) and cortisol, which in turn exert profound psychotropic effects, predominantly anxiety and depressive reactions. Chronic stress also disrupts the regulation of sleep and nutrition, further contributing to the development of depression (Laryea et al. 2012).

### 4.2 HPA Axis Dysfunction

The HPA axis represents the standard stress hormone system that orchestrates the release of glucocorticoids by the adrenal glands in response to environmental or endogenous stressors (Joseph and Whirledge 2017). It serves as a vital interface between the nervous system and the endocrine system, enabling the body to adapt to various stressors (Cain and Cidlowski 2017). Once released, glucocorticoids can function in virtually all parts of the body and regulate a broad range of physiological processes through the genomic and non‐genomic effects of glucocorticoid receptors (GRs) (Stephens and Wand 2012). The HPA axis becomes activated in response to a variety of stressors, such as physical pain, psychological stress, or infections (Leistner and Menke 2020). When activated, the hypothalamus secretes CRH, which stimulates the pituitary gland to release ACTH. ACTH then acts on the adrenal cortex to secrete glucocorticoids, primarily cortisol in humans (Micale and Drago 2018). This cascade of events helps the body to cope with stress by regulating metabolism, immune responses, and neural activity (Bomholt et al. 2004).

The inflammation due to LBP is a key factor in the process through which the pain acts as a stressor and results in the activation of the HPA axis (Kunugi et al. 2010). Inflammatory cytokines, such as interleukin‐1β (IL‐1β), interleukin‐6 (IL‐6), and tumor necrosis factor‐α (TNF‐α), have been shown to promote activation of the HPA axis and elicit glucocorticoid release (Adcock and Glucocorticoids 2017). This inflammatory response not only aggravates the pain but also triggers a cascade of neuroendocrine changes that can impact mood and cognitive function. Glucocorticoids possess both anti‐ and pro‐inflammatory properties (Srinivasan and Walker 2022). Their anti‐inflammatory effects are primarily elicited via GR‐mediated trans‐repression of key inflammatory transcription factors, such as the NF‐κB and activator protein 1 (AP‐1) pathways (Troubat et al. 2021). This results in reduced expression of pro‐inflammatory genes, including chemokines and cytokines, thereby suppressing the inflammatory response (Adcock and Glucocorticoids 2017). The mechanisms underlying these genomic properties involve GR binding to glucocorticoid response factors in the promoter region of pro‐inflammatory genes, negatively modulating AP‐1 transcriptional activity, direct GR interaction with p65 and c‐Jun (subunits of NF‐κB and AP‐1, respectively), as well as recruitment of histone deacetylases to NF‐κB‐dependent promoters. Additionally, glucocorticoids promote tristetraprolin, which destabilizes the mRNA of many pro‐inflammatory cytokines (O'Neill 2008). However, glucocorticoids also exhibit pro‐inflammatory properties under certain conditions. They have been shown to activate Toll‐like receptor (TLR) pathways and increase the expression of several members of the TLR family necessary for inducing inflammation, such as TLR2 and TLR4 (Fietta and Fietta 2007). A synergistic action between glucocorticoids and these cytokines can stimulate pro‐inflammatory pathways, contributing to inflammation and the immune response.

The inflammation associated with LBP, along with the subsequent release of glucocorticoids, can affect the brain in several ways (Colucci‐D'Amato et al. 2020). Chronic exposure to glucocorticoids has been shown to reduce the levels of brain‐derived neurotrophic factor (BDNF) and its receptor, tyrosine kinase receptor B (TrkB), in brain structures involved in depression and the regulation of cognitive and emotional processes (Adcock and Glucocorticoids 2017). BDNF is an important growth factor that supports neuronal survival, differentiation, and synaptic plasticity (Björkholm and Monteggia 2016). Reduced BDNF levels have been implicated in the pathophysiology of depression, contributing to neuronal atrophy and impaired neuroplasticity (Herman et al. 2016). Additionally, glucocorticoids can regulate the negative feedback of the HPA axis, instigating this at multiple levels, directly on the axis components (hypothalamus, pituitary, and adrenal glands) and indirectly via brain structures such as the hippocampus, prefrontal cortex (PFC), and amygdala (Gjerstad et al. 2018). This negative feedback helps to terminate the stress response once the stressor has been removed, thereby maintaining HPA axis homeostasis (Menke 2024). Depression has also been shown to impact the HPA axis. Individuals with depression often exhibit altered HPA axis activity, with increased CRH and cortisol levels indicating HPA axis hyperactivation (Lu et al. 2022). This hyperactivation may be due to impaired GR function or altered sensitivity to glucocorticoids, leading to an inability to terminate the stress response (Harro 2019). The resulting chronic exposure to glucocorticoids can further worsen depression by reducing BDNF levels and impairing neuroplasticity (Suri and Vaidya 2013).

## 5 Neurological Changes

### 5.1 Changes in Brain Structure and Function

Brain imaging studies have illuminated the structural and functional changes occurring in specific brain regions among individuals with chronic LBP (Vlaeyen et al. 2018). Notably, alterations in grey matter volume and connectivity have been seen in several key areas, including the thalamus, dorsolateral PFC (DLPFC), temporal lobes, insula, and the primary somatosensory cortex (Lenz et al. 2004). These findings suggest a complex interaction between nociceptive processing, emotional regulation, and cognitive functioning in chronic LBP. The thalamus, a critical relay station for sensory information, plays a pivotal role in the affective dimension of pain (Vollenweider and Preller 2020). Specifically, the mediodorsal thalamus has been implied to be involved in the emotional processing of painful stimuli. In patients with chronic LBP, disruptions in the coupling between the thalamus and PFC have been documented, highlighting a potential mechanism through which pain perception becomes intertwined with emotional and cognitive disturbances (Linley et al. 2021). Intriguingly, similar thalamic‐PFC coupling deficits have been reported in psychiatric conditions such as depression and schizophrenia, suggesting a shared neurobiological vulnerability (Schmaal et al. 2017).

In depression, the amygdala, a fundamental structure involved in emotional processing and fear conditioning, exhibits increased activity and connectivity. Conversely, other brain regions, such as the subgenual anterior cingulate cortex (sgACC), also show hyperactivity, whereas the insula and DLPFC demonstrate hypoactivity (Culig et al. 2022). These findings reflect a complex pattern of neural activation and deactivation that contributes to the emotional and cognitive symptoms of depression. It is worth noting that the brain changes identified in major depressive disorder (MDD) are highly heterogeneous, reflecting the diverse clinical presentations of this condition. Structural studies on depression have consistently reported smaller hippocampal volumes compared to individuals without depression (Knierim 2015). The hippocampus, a crucial structure for emotional memory processing, is involved in the formation, consolidation, and retrieval of memories (Kim et al. 2017). Notably, alterations in hippocampal volume and function have been linked to memory distortions, including the production of false or incorrect memories and memory interferences (Berger et al. 2018). These memory distortions can significantly impact an individual's perception and interpretation of pain, potentially intensifying the experience of chronic pain. A localized hippocampal circuit, along with personality traits associated with reward processing, has been shown to determine the exaggeration of daily pain experiences in chronic pain patients (Bonilla‐Jaime et al. 2022). This suggests that individual differences in hippocampal functioning and reward sensitivity may contribute to the variability in pain perception and response among individuals with chronic LBP. Adult hippocampal neurogenesis, the process of generating new neurons in the hippocampus, is decreased in both animal models of depression and humans experiencing depressive episodes (Tartt et al. 2022). Conversely, treatment with antidepressant has been shown to induce an increase in adult hippocampal neurogenesis (Du Preez et al. 2021). Experimentally induced increase in hippocampal neurogenesis dampens the ability of stress to induce a depressive‐like phenotype, whereas a decrease in adult neurogenesis alters stress sensitivity (Vasic and Schmidt 2017). Furthermore, ablation of hippocampal neurogenesis prevents the ability of antidepressants to induce remission, highlighting the critical role of hippocampal neuroplasticity in the pathophysiology and treatment of depression.

With regard to chronic LBP, it is plausible that the decreased hippocampal neurogenesis observed in depression may contribute to the persistence and exacerbation of pain symptoms. The reduced capacity for neuroplasticity in the hippocampus may hinder the brain's ability to adapt to and regulate pain, leading to a vicious cycle of chronic pain and depression (Schnellbächer et al. 2022). Moreover, the insula, a brain region involved in interoceptive awareness and emotional processing, shows hypoactivity in depression (Struckmann et al. 2022). Given its role in pain perception and emotional responses to pain, changes in its functioning may further increase the occurrence of chronic LBP among individuals with depression. Similarly, the DLPFC, a key region for executive functioning and cognitive control, also shows hypoactivity in depression, potentially contributing to the cognitive impairments often observed in this condition (Li and Roy 2023).

### 5.2 Decreased BDNF Levels and Decreased Neuroplasticity

BDNF is a protein that plays a crucial role in the brain and nervous system (Numakawa et al. 2010). It is known for its ability to support the survival of existing neurons and encourage the growth and differentiation of new neurons and synapses (Innocenti 2022). This protein is essential for maintaining the health and plasticity of the brain. Neuroplasticity and neurogenesis refer to the brain's ability to change and adapt throughout life (Malhi and Mann 2018). One of the most significant discoveries of this century is the identification of pluripotent stem cells in the adult brain, which can generate new neurons, a process termed neurogenesis (Culig et al. 2022). Neurogenesis is critical for learning, memory, and emotional regulation. It allows the brain to reorganize and form new connections in response to new experiences or injuries (Kim and Park 2021). The impact of neurogenesis on depression is profound. Depression is often associated with decreased neurogenesis, particularly in the hippocampus, a brain region crucial for learning and memory (Kot et al. 2022). Treatments for depression, such as antidepressants and electroconvulsive therapy, have been shown to increase neurogenesis in this area (Giacobbe et al. 2020; Abe et al. 2024). This suggests that enhancing neurogenesis may be a therapeutic strategy for treating depression (Araki et al. 2021). The process of neurogenesis is controlled by regulatory proteins, such as BDNF (O'Leary et al. 2018). In patients with MDD, BDNF levels are often diminished. This reduction in BDNF can impair neurogenesis, leading to decreased neuronal survival and differentiation (Navone et al. 2018). Therefore, increasing BDNF levels may be the key to promoting neurogenesis and alleviating depressive symptoms.

With LBP, the degenerative microenvironment of intervertebral discs (IVDs) can stimulate the release of inflammatory factors, such as IL‐8, NGF, IFN‐γ, and IL‐17 (Cohen et al. 2024). These inflammatory factors drive the activation of microglia in the spinal cord and increase the upregulation of neuroinflammatory markers. This in turn enhances the inflammatory milieu within IVD tissues and in the peridiscal space, aggravating the cascade of degenerative events. Under stress, pain, or pathological conditions, microglia secrete inflammatory mediators that disrupt neuronal function and impair neurogenesis (Zhang et al. 2021). This increases susceptibility to stress, promoting the occurrence and development of depression (He et al. 2020). Microglia, when activated, can either contribute to inflammation and neuronal damage or secrete neuroprotective factors, such as BDNF, depending on their activation state. Interleukin‐4 (IL‐4)‐driven microglia have been shown to synthesize and secrete more BDNF (He et al. 2020). IL‐4 acts as an anti‐inflammatory cytokine that can shift microglia towards a more neuroprotective phenotype (Iannitelli et al. 2021). By increasing BDNF secretion, IL‐4‐driven microglia may promote neurogenesis and neuronal survival, thereby alleviating depressive symptoms associated with LBP.

Gender differences in BDNF secretion are significant (Smith 2024). BDNF derived from microglia plays a major role in males, whereas components of the adaptive immune system, such as invading macrophages and T‐lymphocytes, are predominant in females (Sardar et al. 2021). Across the course of life, depression is almost twice as common in females than in males (Culig et al. 2022). This gender disparity in depression rates may be partially explained by differences in BDNF secretion. Females tend to have lower levels of BDNF compared to males (Salari et al. 2024). This reduction in BDNF may impair neurogenesis and contribute to the higher incidence of depression in females. In addition, women are more susceptible to stress and inflammation (Lamichhane et al. 2021), which can further decrease BDNF levels and exacerbate depressive symptoms. Therefore, gender‐specific approaches to increasing BDNF levels may be necessary to effectively treat depression in females. Thus, the interplay between LBP and depression involves complex mechanisms, including the regulation of neurogenesis by BDNF. By understanding these mechanisms, we can develop more targeted and effective treatments for both conditions.

## 6 Immune‐Metabolic Responses

### 6.1 Intestinal Dysbiosis

Gut dysbiosis refers to a state characterized by the excessive growth of pathobionts (pathological microorganisms) within the gastrointestinal tract (Debnath et al. 2021). This imbalance in microbial populations has been implied to be involved in the pathogenesis of various pathologies, including LBP, cancers, and autoimmune diseases. In a healthy state, the gut microbiome maintains a delicate balance between beneficial and pathogenic microorganisms, playing a crucial role in digestion, immune regulation, and the synthesis of essential nutrients (Rajasekaran et al. 2020). However, when this balance is disturbed, it can lead to a cascade of inflammatory responses and metabolic disturbances, contributing to the development of various diseases.

IVD, which acts as a cushion between the vertebrae in the spine, also harbors a unique microbial community, normally consisting of abundant Firmicutes and Actinobacteria (Ratna et al. 2023). The blood–disc barrier, which surrounds the IVD, safeguards it from systemic infection, resists inflammation, and halts the immune surveillance of its inner aspects (He et al. 2024). However, when this microbial balance within the IVD is disrupted, it can lead to disc degeneration and inflammation, ultimately resulting in LBP. Recent research suggests that gut dysbiosis is not only associated with physical ailments like LBP but also with mental health disorders such as depression. The growing body of data indicates that probiotics can be used as an effective treatment for mental disorders where increased intestinal permeability has been demonstrated, including depression, anxiety, autism, schizophrenia, and bipolar disorder. The mode of action of these probiotic microorganisms includes regulation of the immune system, production of short‐chain fatty acids (SCFAs), and support of the gut barrier integrity.

The gut–brain axis, a bidirectional communication system between the gastrointestinal tract and the central nervous system (CNS), further outlines the link between gut dysbiosis and mental health. Changes in the gut microbiome can influence neurotransmitter levels, immune function, and HPA axis activity, all of which play critical roles in mood regulation and stress response.

Regarding LBP and depression, specific gut microbiome changes have been identified. For instance, research has demonstrated that individuals with chronic LBP often have an imbalance in their gut microbiome, with a reduction in beneficial bacteria such as *Lactobacillus* and *Bifidobacterium* and an increase in pathogenic bacteria like *Escherichia coli* and *Pseudomonas*. Similarly, patients with depression have been found to have altered gut microbiome profiles, characterized by increased levels of inflammatory bacteria and decreased levels of neuroprotective bacteria. Several animal models have been used to investigate the role of gut dysbiosis in the development of LBP and depression. As an example, studies using chronic restraint stress models in rats have shown that stress‐induced gut microbiome alterations can lead to depressive‐like behaviors and increased sensitivity to pain. Additionally, animal models of induced disc degeneration have demonstrated that changes in the gut microbiome can exacerbate disc inflammation and pain. However, these animal models are not without their limitations. For instance, chronic restraint stress models may not fully capture the complex interplay between psychological, social, and biological factors that contribute to human depression. Similarly, induced disc degeneration models may not accurately reflect the natural history and progression of LBP in humans.

### 6.2 Chronic Inflammation

Inflammation is a complex biological response of body tissues to harmful stimuli, such as infection, injury, or irritation. LBP is a prevalent condition that can arise from various sources, including mechanical stress, degenerative changes, and injuries (Klyne et al. 2022). The inflammatory response in LBP is characterized by the release of several cytokines and acute‐phase reactants (Khan et al. 2017). High‐sensitivity C‐reactive protein (CRP) is one of the first described acute‐phase proteins and serves as a systemic marker of inflammation (Pinto et al. 2023). Furthermore, cytokines such as TNF‐α, soluble TNF receptor 1 (sTNFR1), and IL‐6 have been identified as potential regulators of different aspects of acute LBP (Dantzer 2006). These inflammatory cytokines, once produced in the periphery due to LBP, can gain access to the brain through multiple pathways. One such pathway is the neural route, through which primary afferent neurons innervating the site of inflammation transmit signals to the brain. Another route is the humoral pathway, which involves the production of pro‐inflammatory cytokines by phagocytic cells in the circumventricular organs (CVOs) and choroid plexus in response to circulating pathogen‐associated molecular patterns (PAMPs) or cytokines (Liu et al. 2012). These immune signals then propagate into the brain parenchyma.

Once these inflammatory cytokines enter the brain, they can initiate neuroinflammation primarily through the activation of microglia, the resident immune cells of the CNS. Cytokine release induced by stressors penetrates the blood–brain barrier and activates microglia via IL‐6 receptors or TNF receptors (Taylor et al. 2010). Activated microglia can affect neural progenitor cells, inhibiting their proliferation and differentiation (Harada et al. 2019). Furthermore, IL‐6 promotes monocyte differentiation into macrophages and lymphocyte maturation (Wagner and Myers 1996), thereby mediating the acute‐phase response to injury. TNF‐α, on the other hand, stimulates inflammatory responses and induces nerve swelling and neuropathic pain, as well as promotes cellular apoptosis through its cytotoxic effect (Bairamian et al. 2022). Neuroinflammation refers to the inflammatory response within the CNS, characterized by the activation of microglia and astrocytes, which leads to the release of pro‐inflammatory cytokines, reactive oxygen species, and nitric oxide. Microglia play a pivotal role in sensing environmental changes and responding to harmful stimuli, as well as phagocytosing debris and apoptotic neurons (Kwon and Koh 2020). With regard to neuroinflammation, microglia release cytokines that can further activate astrocytes and neurons, creating a feedback loop that perpetuates the inflammatory state (Ly et al. 2023). The neuroinflammatory process has been associated with the pathogenesis of depression (Rodríguez‐Palma et al. 2024). Several meta‐analyses have consistently reported increased levels of pro‐inflammatory cytokines and acute‐phase proteins, such as IL‐6, TNF‐α, and CRP, in the blood of patients with MDD compared to healthy controls (Shorey et al. 2022). Clinical depression and sickness behavior, characterized by symptoms such as malaise, hyperalgesia, pyrexia, disinterest in social interactions, lethargy, and anhedonia, are highly connected phenotypes that are mediated by pro‐inflammatory cytokines like IL‐1, IL‐6, and TNF‐α. Furthermore, whether neuroinflammation is associated with neuro‐related diseases such as anxiety, irritability, cognitive dysfunction, and Alzheimer's disease also requires more in‐depth research.

Animal models have been instrumental in studying the relationship between inflammation, LBP, and depression. For instance, rodent models of chronic pain induced by spinal nerve ligation or disc herniation have shown increased levels of pro‐inflammatory cytokines in the CNS and behavioral symptoms resembling depression. However, these models have limitations, such as the inability to fully replicate the complexity of human pain and depression, as well as the potential species‐specific differences in inflammatory responses (Korah et al. 2022; Palta et al. 2014).

### 6.3 Cell‐Level Damage

LBP and depression are two prevalent conditions that heavily affects individuals’ quality of life. There is emerging evidence suggesting a potential link between these two conditions, particularly through the mechanism of oxidative stress (Hashmi et al. 2022). The lumbar spine is composed of five vertebral bones, interconnected by IVD, which act as shock absorbers and facilitate movement (Kamali et al. 2021). Among the various causes of LBP, MDD stands out as a major contributor, accounting for approximately 40% of symptomatic LBP cases (Lama et al. 2013). As discs degenerate, they lose water content, decrease in elasticity, and narrow the disc space, conceivably leading to herniated discs and spinal stenosis (Good 2017). ROS, including superoxide anion (O_2_^−^), hydroxyl radical (OH^−^), hydrogen peroxide (H_2_O_2_), and hypochlorite ion (OC1^−^), are unstable and highly reactive molecules produced as by‐products of cellular aerobic metabolism (Herb and Schramm 2021). At normal levels, ROS function as important intracellular signal molecules, participating in the regulation of various physiological processes (Singh et al. 2019). However, excessive ROS generation, coupled with the exhaustion of antioxidative defense, triggers pro‐inflammatory signaling, thus damaging vital macromolecules and inducing cellular apoptosis (Wang et al. 2023). In degenerative IVDs, a decline in the recycling capacity of substances and various stress stimuli create a hostile microenvironment (Zorov et al. 2014). This leads to mitochondrial dysfunction, impairing mitochondrial dynamics and quality control systems, thereby increasing ROS production (Suzuki et al. 2015). Studies by Suzuki et al. have demonstrated that the level of ROS in human and rat IVDs gradually elevates with increasing grades of IVD degeneration (Bhatt et al. 2020). This ROS overproduction exacerbates the degenerative process, contributing to further tissue damage and pain (Zorov et al. 2014). The brain, due to its higher oxygen consumption, higher lipid content, and weaker antioxidative defense, is particularly vulnerable to oxidative stress. ROS have vital roles in cellular signaling and in defense against invasive microorganisms (Angelova and Abramov 2018). However, when ROS generation exceeds the antioxidant capacity, it disrupts redox homeostasis, leading to cell necrosis and neurodegeneration (Culmsee et al. 2019). Oxidative stress is a main cause of neurodegeneration, and its involvement in the pathogenesis of MDD is unequivocally established (Angelova and Abramov 2018). The damage of cell membrane lipid bilayers will lead to abnormal transmembrane exchanges of various molecules, ions, and so forth, disrupting physiological and biochemical homeostasis within the cell and consequently causing cell damage. Brain neurons, astrocytes, and microglia are rich in mitochondria and NADPH oxidase (NOX), capable of generating abundant ROS (Fu et al. 2010). Studies found significantly reduced antioxidants (e.g., SOD, GSH) and increased peroxidation biomarkers, such as carbonyl and malondialdehyde (MDA), in depressed patients and animals (Parul et al. 2021). Animal studies further specified stress‐induced abnormalities across brain subregions, such as the reduced GSH and SOD activity and increased levels of ROS, MDA, and carbonyl in the PFC and hippocampus of depressed rats subjected to chronic unpredictable mild stress (CUMS), chronic restraint stress, and chronic social isolation, respectively (Juruena et al. 2020).

**FIGURE 1:**
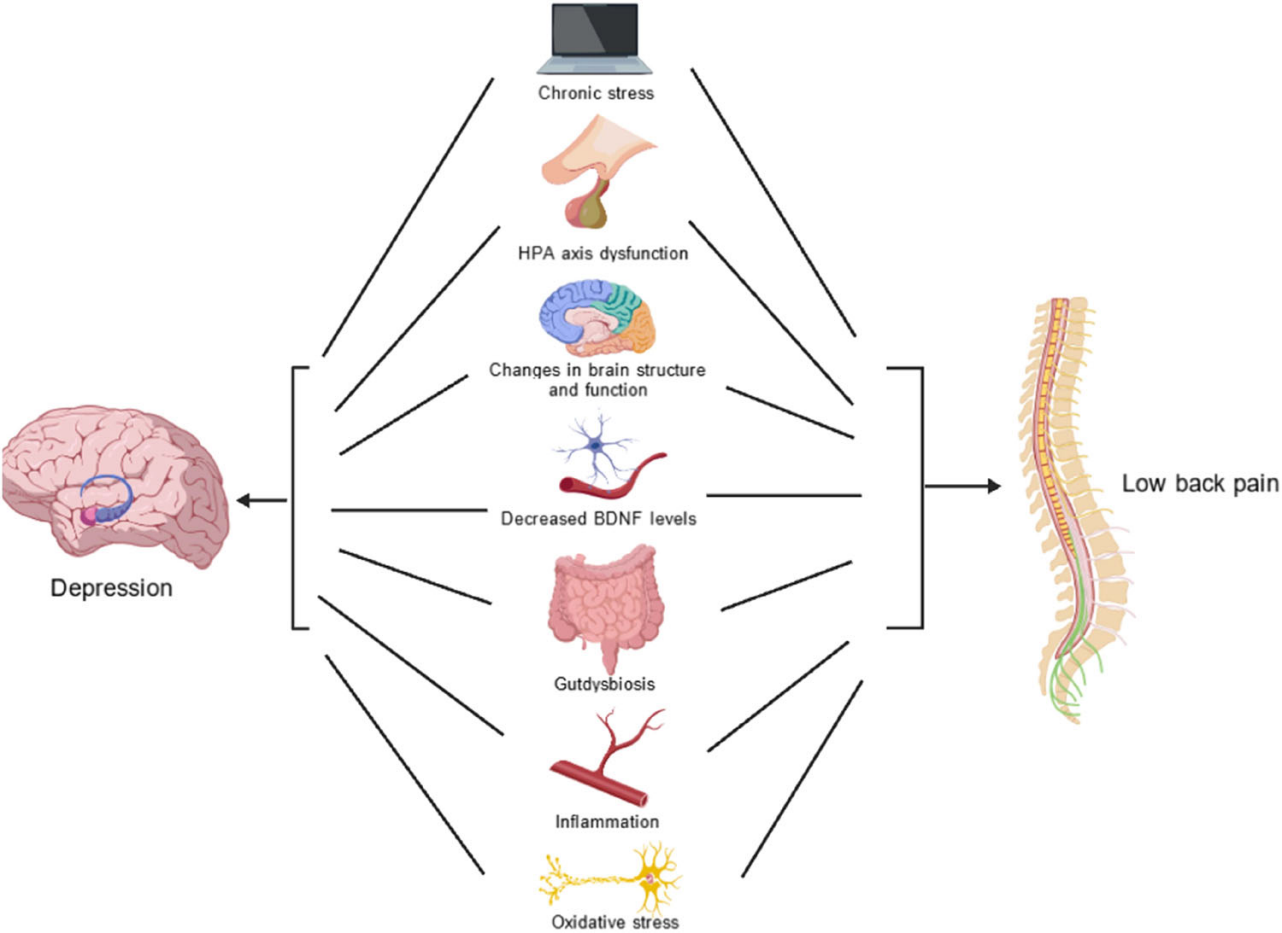
The potential relationship between low back pain and depression. BDNF, brain‐derived neurotrophic factor; HPA, hypothalamic‐pituitary‐adrenal.

Given the established roles of ROS in both IVD degeneration and depression, it is plausible to hypothesize that oxidative stress might serve as a common underlying mechanism between LBP and depression. The degenerative process in IVDs releases ROS, contributing to tissue damage, inflammation, and pain. Simultaneously, oxidative stress in the brain disrupts neuronal function, leading to neurodegeneration and depression. Although this hypothesis requires further investigation, it opens new avenues for potential therapeutic interventions that target oxidative stress to alleviate both LBP and depression. The potential relationship between LBP and depression is shown in Figure 1.

### 6.4 Therapy

Current pharmacological management of LBP prioritizes NSAIDs and anticonvulsants, whereas antidepressants (e.g., SSRIs/SNRIs) are first‐line for depression. Evidence indicates tricyclic/tetracyclic antidepressants (TCAs/TeCAs) significantly improve LBP symptoms in depressed patients, though efficacy appears independent of depressive status (Staiger et al. 2003). Their analgesic mechanism in nonmalignant pain may stem from intrinsic neurochemical properties, potentially involving monoamine modulation (Onghena and Van Houdenhove 1992). Concurrently, anti‐inflammatory agents show antidepressant benefits in MDD, suggesting bidirectional neuroimmune interactions (Bai et al. 2020). However, precise mechanisms remain unclear. Further investigation into shared pathways—such as HPA axis dysregulation, neuroinflammation, and oxidative stress—is warranted to elucidate the LBP‐depression comorbidity and optimize targeted therapies.

Physical therapy (PT), encompassing structured exercise, manual techniques, and neuromodulation, is integral to managing comorbid LBP and depression. Evidence demonstrates PT significantly alleviates LBP symptoms (e.g., 16‐week core stabilization programs reduce pain intensity, *p = 0.040*) and improves depressive affect (BDI‐II somatic scores decrease, *p = 0.042*) (Hušáková et al. 2023). Complementary approaches like Tai Chi enhance functional mobility, psychological resilience, and social support in chronic LBP patients (Zhang et al. 2023). Adjunctive lifestyle modifications—reducing alcohol/tobacco use, optimizing sleep, and limiting digital overuse—further mitigate depression risks linked to these behaviors in both genders (Sunderland et al. 2021). Critically, addressing psychological factors is essential, as 30% of chronic LBP patients exhibit clinical depression (Manchikanti et al. 2002).

Psychological interventions tailored to patient profiles show robust efficacy: cognitive behavioral therapy (CBT) targets maladaptive cognitions, whereas acceptance and commitment therapy (ACT) improves pain acceptance and reduces functional impairment (ACT is superior to controls in pain/depression outcomes). Behavioral activation (a CBT variant) rivals classical CBT in depressive symptom reduction (Sanabria‐Mazo et al. 2020). Interpersonal therapy (IPT) resolves relational stressors, and mindfulness‐based cognitive therapy (MBCT) prevents relapse in recurrent depression. However, multidisciplinary biopsychosocial rehabilitation—combining PT, psychological therapy, and medical care—outperforms unimodal PT. Meta‐analyses confirm its superiority for long‐term pain relief (SMD 0.21) and disability reduction (SMD 0.23), underscoring the synergy of integrated approaches (Kamper et al. 2015).

## 7 Discussion

There exists a complicated and profound correlation between LBP and depression. The onset of this pathological process often stems from chronic stress triggered by external pressures. As this process deepens, it gradually affects not only the nervous and immune systems but also reaches down to the cellular level. During this process, chronic stress activates the HPA axis, whereas prolonged states of LBP and depression lead to significant abnormalities in brain structure and function and fluctuations in the levels of BNDF. Meanwhile, an imbalance in gut microbiota further exacerbates chronic inflammation, which happens to be a crucial link between LBP and depression. Under the interplay of multiple factors, oxidative stress gradually intensifies, causing cellular damage and thereby worsening the symptoms of LBP and depression. This elaborate series of mechanisms collectively paints a complex picture of the comorbidity of LBP and depression.

On the other hand, when utilizing AI for writing, we must also acknowledge its limitations. Although AI can efficiently generate text, it still struggles when dealing with complicated medical issues. Especially in the absence of sufficient literature supplements and relevant knowledge, AI's descriptions often fall short of accuracy and may even contain errors. In such cases, manual correction and further guidance for AI are required. In addition, due to AI's lack of deep understanding of human emotions, experiences, and individual differences, it struggles to fully capture all the nuances and complexities of the comorbidity of LBP and depression. Therefore, in medical research, it is still necessary to combine researchers’ extensive literature reading, scientific research experience, and profound understanding of human emotions to further refine and enhance the content written by AI.

## 8 Conclusion

The potential relationship between LBP and depression represents a fascinating and complex topic of research that holds significant implications for patient care and treatment outcomes. By continuing to explore the underlying mechanisms and developing targeted interventions, we can move closer to addressing the needs of this vulnerable population as well as improving their overall quality of life. Moreover, it is feasible to assist writing by combining AI tools with writing methods, indicating that AI can be used to assist researchers in scientific research in the future.

## Author Contributions

**Fushui Liu**: writing – review and editing, project administration, investigation, resources, funding acquisition. **Li Cao**: writing – review and editing, writing – original draft. **Biao Deng**: writing – review and editing, data curation. **Xiaole Wang**: data curation. **Ting Fang**: data curation. **KuyikAbasi Nsima Umoh**: data curation. **Lang Liu**: data curation. **Jiaming Qian**: data curation.

## Funding

The study was supported by the National Natural Science Foundation of China [No. 82360940] and the University Leading Talents of Ganpo Tanlents Project.

## Ethics Statement

We confirm that we have read the journal's position on issues involved in ethical publication and affirm that this report is consistent with those guidelines.

## Consent

The authors have nothing to report.

## Conflicts of Interest

The authors declare no conflicts of interest.

## Peer Review

The peer review history for this article is available at https://publons.com/publon/10.1002/brb3.71026.

## Data Availability

The authors have nothing to report.
